# Impairment of Social-Related Quality of Life in COVID-19 Pneumonia Survivors: A Prospective Longitudinal Study

**DOI:** 10.3390/jcm12247640

**Published:** 2023-12-12

**Authors:** Takahiro Ando, Sho Shimada, Jun Sugihara, Koji Takayama, Masayoshi Kobayashi, Yoshihiro Miyashita, Tatsuya Ito, Kaori Okayasu, Shun Tsuyuki, Takehiko Ohba, Masafumi Doi, Hiroaki Saito, Toshihide Fujie, Tomoshige Chiaki, Atsushi Nakagawa, Tatsuhiko Anzai, Kunihiko Takahashi, Sho Shibata, Tomoya Tateishi, Yasunari Miyazaki

**Affiliations:** 1 Department of Respiratory Medicine, Tokyo Medical and Dental University, 1-5-45 Yushima, Bunkyo-ku, Tokyo 113-8519, Japan; andopulm@tmd.ac.jp (T.A.); shi2pulm@tmd.ac.jp (S.S.); sugihara.pulm@tmd.ac.jp (J.S.); shibata.pulm@tmd.ac.jp (S.S.); miyazaki.pilm@tmd.ac.jp (Y.M.); 2 Department of Respiratory Medicine, Musashino Red Cross Hospital, 1-26-1 Kyonancho, Musashino-shi, Tokyo 180-8610, Japan; kojita.pulm@gmail.com; 3 Department of Respiratory Medicine, Tokyo Metropolitan Bokutoh Hospital, 4-23-15 Koutoubashi, Sumida-ku, Tokyo 130-8575, Japan; masayoshi_kobayashi@tmhp.jp; 4 Department of Respiratory Medicine, Yamanashi Prefectural Central Hospital, 1-1-1 Fujimi, Kofu-shi, Yamanashi 400-8506, Japan; y-miyashita@ych.pref.yamanashi.jp; 5 Department of Respiratory Medicine, Yokosuka Kyosai Hospital, 1-16 Yonegahama-dori, Yokosuka-shi, Kanagawa 238-8558, Japan; tatuya.ito.110@gmail.com; 6 Department of Respiratory Medicine, Yokohama Municipal Minato Red Cross Hospital, 3-12-1 Shinyamashita, Naka-ku, Yokohama-shi, Kanagawa 231-8682, Japan; k.okayasu.pulm@gmail.com; 7 Department of Respiratory Medicine, Kudanzaka Hospital, 1-6-12 Kudanminami, Chiyoda-ku, Tokyo 102-0074, Japan; tsuyuki.s@gmail.com; 8 Department of Respiratory Medicine, Ome Municipal General Hospital, 4-16-5 Higashi-ome, Ome-shi, Tokyo 198-0042, Japan; ohba-ta@mghp.ome.tokyo.jp; 9 Department of Respiratory Medicine, Kashiwa Municipal Hospital, 1-3 Fuse, Kashiwa-shi, Chiba 277-0825, Japan; swiftmove@hotmail.co.jp; 10 Department of Respiratory Medicine, Tsuchiura Kyodo General Hospital, 4-1-1 Otsuno, Tsuchiura-shi, Ibaraki 300-0028, Japan; saitou2133@tkgh.jp; 11 Department of Respiratory Medicine, Tokyo Metropolitan Ohtsuka Hospital, 2-8-1 Minami-ohtsuka, Toshima-ku, Tokyo 170-8476, Japan; toshihide_fujie@tmhp.jp; 12 Department of Respiratory Medicine, Hokushin General Hospital, 1-5-63 Nishi, Nakano-shi, Nagano 383-8505, Japan; resp-hgh@hokushin-hosp.jp; 13 Department of Respiratory Medicine, Tokyo Kyosai Hospital, 2-3-8 Nakameguro, Meguro-ku, Tokyo 153-8934, Japan; achiko55@cameo.plala.or.jp; 14 Department of Biostatistics, M&D Data Science Center, Tokyo Medical and Dental University, 1-5-45 Yushima, Bunkyo-ku, Tokyo 113-8519, Japan; tanzai.dsc@tmd.ac.jp (T.A.); kunihikot.dsc@tmd.ac.jp (K.T.)

**Keywords:** COVID-19 pneumonia, post-acute sequelae of SARS-CoV-2 infection, respiratory sequelae, quality of life, SF-36

## Abstract

The post-acute sequelae of SARS-CoV-2 (PASC) pose a threat to patients’ health-related quality of life (HRQOL). Here, the impact of COVID-19 on HRQOL and the clinical factors associated with impaired HRQOL were examined. Discharged COVID-19 patients were assessed at 3 and 6 months after disease onset. The patients completed a medical examination and the SF-36 questionnaire at these two time points and underwent pulmonary function testing at 6 months after disease onset. All had undergone computed tomography (CT) imaging upon hospital admission. Of the 74 included patients, 38% reported respiratory symptoms at 3 months, and 26% reported respiratory symptoms at 6 months after disease onset. The aggregated SF-36 scores declined in the role/social component summary (RCS), a category related to social activity. Patients with lower RCS tended to have respiratory sequelae or a relatively lower forced vital capacity. The CT score that reflected the extent of COVID-19 pneumonia was inversely correlated with the RCS score (3 months, *p* = 0.0024; 6 months, *p* = 0.0464). A high CT score (≥10 points) predicted a low RCS score at 6 months (*p* = 0.013). This study highlights the impairment of RCS and its associations with respiratory sequelae. The study also emphasizes the importance of radiological findings in predicting long-term HRQOL outcomes after COVID-19.

## 1. Introduction

Coronavirus disease 2019 (COVID-19), caused by severe acute respiratory syndrome coronavirus 2 (SARS-CoV-2), is a disease that caused a global pandemic, which, for some parts of the world, is still ongoing. As of 20 October 2023, more than 677 million people worldwide have been infected with SARS-CoV-2, with more than 6.9 million deaths being attributed to the disease [1] [accessed 20 October 2023]. Mortality rates have decreased due to a combination of factors, including the widespread use of vaccines, treatment with antiviral drugs [2], and changes in viral strains [3]. However, the long-term sequelae that occur in COVID-19 survivors have become a growing concern.

The post-acute sequelae of SARS-CoV-2 infection (PASC), according to the World Health Organization, are characterized by the persistence of symptoms or new symptoms more than 30 days after SARS-CoV-2 infection [4]. Various symptoms of PASC, such as fatigue, dyspnea, anxiety/depression, palpitations, and hair loss [5], have been reported, and these symptoms can last for more than 6 months in some patients [6]. Factors that may contribute to PASC include being female, a history of smoking, a high body mass index (defined as ≥30 kg/m^2^), comorbidities, and intensive care unit admission in the acute phase [7]. In particular, patients with COVID-19 pneumonia have been reported to experience a long-term decline in respiratory function and fibrosis of the lungs [8]. These sequelae and respiratory function impairments can seriously impact a patient’s quality of life (QOL); thus, they need to be identified and addressed from both a medical and social perspective.

Measuring health-related quality of life (HRQOL) is useful for obtaining a comprehensive assessment of a patient’s health status. Assessing HRQOL can help determine the burden of preventable disease, injuries, and disabilities and can provide valuable insight into the relationships between HRQOL and risk factors [9]. The 36-item Short-Form Health Survey (SF-36) is a scientifically validated and reliable multidimensional scale that has been established as a questionnaire to measure HRQOL [10].

Previously reported studies that have assessed PASC at various time points after disease onset have found a decrease in SF-36 scores [11,12,13,14,15,16,17,18]. However, the recovery process of HRQOL after COVID-19 is still not well understood. In addition, the factors associated with the prolonged impairment of HRQOL are also unclear.

This study aimed to systematically identify the trajectories of HRQOL and elucidate the predictors associated with low HRQOL in COVID-19 survivors.

## 2. Materials and Methods

### 2.1. Study Design and Participants

This was a multicenter prospective longitudinal cohort study that followed up hospitalized COVID-19 patients after discharge. The participants were previously hospitalized patients who visited an outpatient clinic 3 months after disease onset and gave consent for their participation in this study. Patients whose health condition improved were not forced to visit an outpatient clinic. The participants were recruited from 10 hospitals in Japan (1 university hospital and 9 general hospitals) from 1 April 2020 to 31 December 2021.

Patients over 20 years of age who were (1) diagnosed with COVID-19 based on SARS-CoV-2 PCR test results, (2) required hospitalization, and (3) were discharged alive were included in this study. Patients who (1) did not visit an outpatient clinic, (2) did not submit a completed version of the SF-36 questionnaire, and (3) had missing data were excluded.

Written informed consent was obtained from all participants.

### 2.2. Procedures

The baseline characteristics and status during hospitalization (disease severity, treatment status, and chest computed tomography (CT) severity score on admission) of each patient were obtained from patients’ medical records. Patients were assessed at 3 and 6 months after COVID-19 onset. During the visits, the patients were interviewed about PASC. Based on patients’ complaints, the symptoms were aggregated by dyspnea, cough, fatigue, numbness, joint pain, olfactory disorder, diarrhea, and fever. Dyspnea, cough, or fatigue were defined as respiratory-related symptoms in this study. At both time points, each participant underwent a physical examination and filled out the SF-36 questionnaire. A pulmonary function test was administered at 6 months after disease onset.

## 3. Measures

### 3.1. SF-36 Questionnaire

The SF-36 questionnaire consists of 36 questions measuring eight domains related to HRQOL. The eight component scores relate to physical function (PF), role physical (RP), bodily pain (BP), general health (GH), vitality (VT), social functioning (SF), role emotional (RE), and mental health (MH). The eight domains are also aggregated into three summary measures: the physical component summary (PCS) score, mental component summary (MCS) score, and role/social component summary (RCS) score [19]. In this study, the eight component scores and component summary scores were calculated as the deviation from the Japanese national standard of 50 points [20,21]. A low score (less than 50 points) is indicative of poor HRQOL.

### 3.2. Chest CT Evaluation

Patients underwent chest CT scans while in the supine position and holding their breath following inspiration. In our study, two pulmonologists with 11 years and 12 years of experience independently assessed the images of patients at admission. The pulmonologists were blinded to the clinical information and clinical course of the patients, except for the knowledge that these images were of patients with COVID-19. The pneumonia CT scores of the patients were recorded using the method described by Chang et al. [22]. In this method, each of the five lung lobes is visually scored on a scale of 0 to 5 points according to the extent of lobar involvement (0: no involvement, 1: involvement of less than 5%, 2: 5–25% involvement, 3: 26–49% involvement, 4: 50–75% involvement, 5: involvement of more than 75%). In cases where there was a discrepancy between the two pulmonologists, the final scores were determined by consensus. Each patient’s total CT score was calculated by adding each lobar score (ranging from 0 to 25 points). CT score was classified into two groups based on cutting off according to the median value.

### 3.3. Pulmonary Function Tests

Spirometry was conducted to measure the forced vital capacity (FVC) and the forced expiratory volume in 1 s (FEV1). The FEV1/FVC ratio was then calculated. The pulmonary function test procedures were conducted in accordance with the American Thoracic Society and European Respiratory Society guidelines [23,24]. The results are expressed as liters and percentages.

## 4. Data Analysis

In this descriptive analysis, continuous variables are expressed as the mean and standard deviation, and categorical variables are expressed as numbers and percentages. Student’s *t*-test was used to compare the means of continuous variables, while simple linear regression analysis was used to compare continuous variables. Multiple linear regression analysis was performed to identify independent predictors associated with low HRQOL. The statistical analysis was conducted using EZR software version 1.54 (Saitama Medical Center, Jichi Medical University, Saitama, Japan) [25], and GraphPad Prism version 9.4.1 (GraphPad Software Inc., La Jolla, CA, USA) was used for graphics visualization. Statistical significance was considered to be *p* < 0.05.

## 5. Results

**Figure 1:**
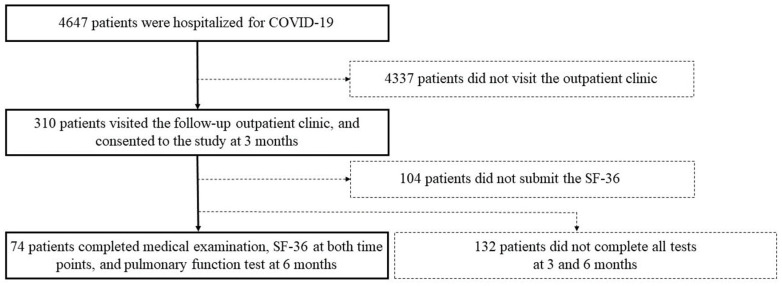
Flow chart of the study from 1 April 2020 to 31 December 2021. This study was conducted among 74 COVID-19 survivors who completed all tests at 3 and 6 months after disease onset.

From 1 April 2020 to 31 December 2021, 4647 patients were hospitalized for COVID-19 in our facilities. Among the patients who visited a follow-up outpatient clinic 3 months after disease onset, 310 patients consented to participate in this study. Out of the 310 patients, we analyzed 74 patients who completed the physical examination and SF-36 questionnaire at the two time points and the pulmonary function test at the 6-month follow-up (Figure 1). In this cohort, 46% were over 65 years old, 73% were male, and 65% had a history of smoking. A total of 88% received medication during hospitalization; 65% were treated with systemic steroids, and 34% were treated with remdesivir. Overall, 80% received supplemental oxygen, and 19% were intubated. Additional baseline characteristics are described in Table 1.

**Figure 2:**
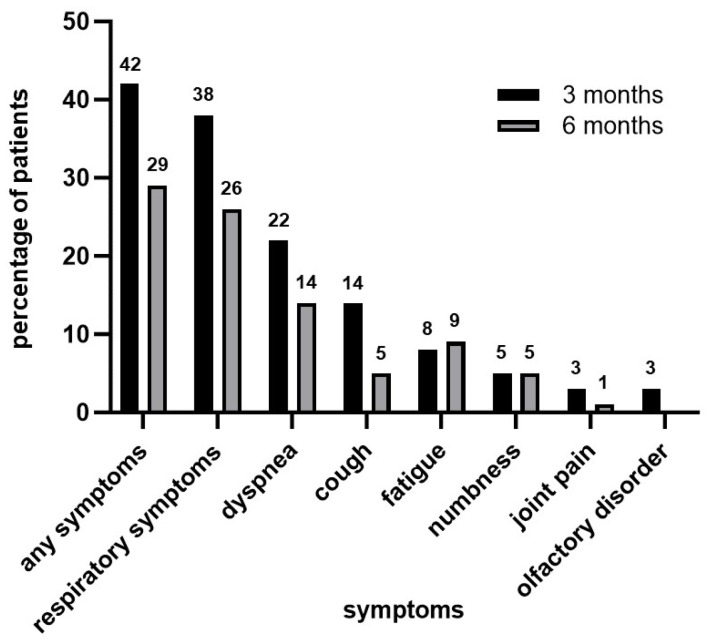
Symptoms observed at 3 and 6 months after COVID-19 onset.

At 3 months after disease onset, 31 patients (42%) reported at least one symptom, and the most common symptoms were dyspnea (22%), cough (14%), and fatigue (8%). At 6 months after disease onset, there was an overall trend toward symptom improvement; however, 21 patients (29%) still experienced persistent symptoms. The most common residual symptoms were dyspnea (14%), cough (5%), and fatigue (9%) (Figure 2). Diarrhea and fever are not shown in Figure 2 because no participants had these symptoms.

The eight component scores and three component summary scores normalized by the Japanese national standard at each time point are shown in Table 2. At 3 months after disease onset, the average scores of five components (PF, RP, BP, SF, and RE) of the SF-36 were below the Japanese national standard (50 points). The average scores of PF and BP, which are the main components of PCS, were slightly below the national standard and had minimal change from 3 to 6 months. On the other hand, the average scores of RP, SF, and RE, the main components of RCS, were the three lowest component scores among the eight components at 3 months after disease onset. These three component scores showed recovery at 6 months after disease onset. Reflecting on these results, RCS was the lowest of the three component summary scores at 3 months, but an improvement in the average scores was observed upon follow-up after 6 months.

We further investigated the association between PASC and low RCS score at each time point. While a statistically significant association was not observed between each PASC and RCS, patients with residual respiratory-related symptoms (dyspnea, cough, or fatigue) tended to have a low RCS score at 3 months (*p* = 0.037). In addition, although not statistically significant, the RCS score tended to be lower in patients who had any symptoms (*p* = 0.090), respiratory-related symptoms (*p* = 0.088), or dyspnea symptoms (*p* = 0.086) at 6 months. Diarrhea and fever were not analyzed because no participants had these symptoms at either time point (Table 3). Several pulmonary function tests such as FVC and FEV1 (measured at the 6-month follow-up) were also significantly associated with the RCS score (Table 3).

**Figure 3:**
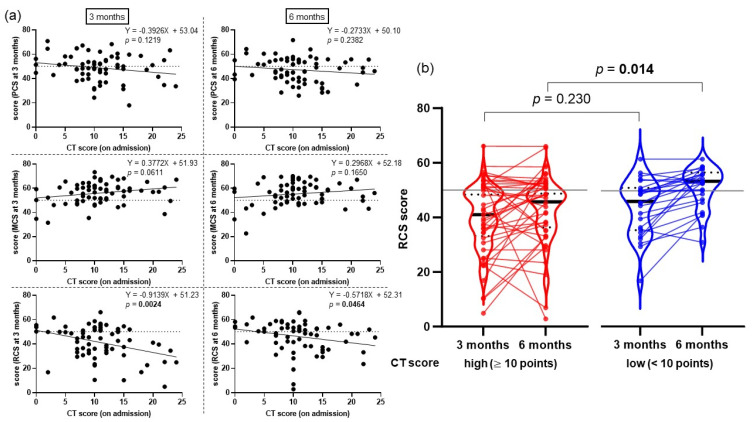
(**a**) Association between CT score and component summary score. Comparisons were determined by simple regression analysis. (**b**) RCS score trajectories from 3 to 6 months of two groups based on CT score (cut-off = median value of 10 points). Groups with high CT scores (≥10 points) are shown in red and groups with low CT scores (<10 points) are shown in blue. The gray bar shows the Japanese national standard (50 points). Comparisons were determined by Student’s *t*-test. For both figures, statistically significant differences (*p* < 0.05) are in bold. Abbreviations: PCS, physical component summary; MCS, mental component summary; RCS, role/social component summary.

We conducted a univariate analysis to examine whether the clinical history and disease course in the acute phase of illness may have influenced the RCS score. Age (over 65 years) was related to a low RCS score at both time points (3 months: *p* = 0.016; 6 months: *p* = 0.016). The use of a high-flow nasal cannula and a high CT score on admission (median value ≥ 10 points) were associated with a low RCS score at 3 months (*p* = 0.007) and 6 months (*p* = 0.014), respectively (Table 4). The association between CT score on admission and a low RCS score was also confirmed by the significant negative linear relationship between the CT score and the RCS score at both time points, while no relation between the CT score and the PCS or MCS score was observed (Figure 3a). Concerning the trajectory of the RCS score, the group with high CT scores showed poorer recovery (average of score change between 3 and 6 months: 3.4 points) than the group with low CT scores (average of score change between 3 and 6 months: 7.1 points) (Figure 3b). A multiple regression analysis that included parameters such as age (older than 65 years), sex (male), smoking history, intubation, history of respiratory disease, and CT score (median value ≥ 10 points) was conducted. These parameters were selected based on the risk factors of PASC elucidated in previous studies [7,26]. Also, we included CT score since it was a factor that was significantly associated with low RCS in the univariate analysis. The results showed that a history of intubation (*p* = 0.031) was independent of a low RCS score at 3 months, and a high CT score (median value ≥ 10 points) (*p* = 0.013) was independent of a low RCS score at 6 months (Table 5).

## 6. Discussion

In this Japanese COVID-19 prospective cohort study, we observed a decline in HRQOL that was especially related to patients’ RCS scores. A decline in RCS score was strongly associated with respiratory symptoms and impaired pulmonary function. Although the average RCS score improved from 3 to 6 months after disease onset, the score had still not reached the Japanese national standard at the 6-month follow-up, indicating that the recovery of the RCS score was insufficient at this time point. Furthermore, patients with a high CT score had a significantly lower RCS score at 6 months. These findings could be useful in predicting long-term low RCS scores.

Several cross-sectional and longitudinal studies evaluating HRQOL have found that HRQOL is often impaired in patients with PASC. The largest Japanese cohort study on sequelae after COVID-19 revealed significantly lower HRQOL scores in the symptomatic group after 3, 6, and 12 months of follow-up [27]. Van den Borst et al. evaluated HRQOL in patients with mild to critical disease at 3 months after onset and reported an overall decline (particularly in RP) in HRQOL scores [17]. O’Brien et al. followed COVID-19 patients at 6 and 12 months after onset and found a decline in HRQOL scores. In particular, RP and RE were found to decline in patients who had not recovered in terms of physical function [12]. Other studies also reported that the categories of RP, SF and RE which comprise the RCS score, were particularly low in PASC patients [14,15]. Based on these studies, HRQOL related to social activities was found to be lower in patients with PASC. These trends were also validated in longitudinal studies. A cohort study following patients who suffered from COVID-19 pneumonia found marked declines in RP, SF, and RE at the 3-month follow-up, but these were improved after 12 months of follow-up [28]. In this study, we also observed declines in RP, SF, and RE. Meanwhile, RCS also showed recovery trends at 6 months in our cohort. These trends are consistent with the above previous studies, and RCS may represent aspects of HRQOL affected by PASC. These components may be indicators that reflect the impact of PASC on HRQOL.

Previous studies have already reported that patients with dyspnea and fatigue have lower HRQOL scores after COVID-19 [29]. In this study, a low RCS score was significantly or nearly significantly associated (3 months; *p* = 0.037, 6 months; *p* = 0.088) with PASC-related respiratory symptoms at both time points. Decreased pulmonary function was observed in patients with lower RCS scores at the 6-month follow-up (FVC: *p* = 0.028, FEV1: *p* = 0.037). Despite the fact that our study protocol, which involved identifying symptoms based on open-ended questions, may have led to some omissions regarding the symptoms, these trends suggest that the respiratory-related problems observed in PASC may influence social-related QOL.

The relation between respiratory symptoms in other chronic respiratory diseases and the components of the RCS score has been previously investigated. In patients with chronic obstructive pulmonary disease, several SF-36 component scores (including RP and SF) were significantly impaired in patients with a low baseline dyspnea index score [30]. In patients with interstitial pneumonia, the observed respiratory symptoms and severely impaired pulmonary function were associated with a decline in overall HRQOL, including in the categories related to social activities [31]. This commonly observed result in chronic respiratory diseases possibly reflects patients’ decreased activity and interaction with others because of their symptoms. Moreover, patients with PASC may avoid social interactions due to concerns about being judged by others because of their prolonged respiratory symptoms.

In this study, having a history of intubation was a predictor of low RCS at the 3-month follow-up, a finding that is consistent with previous reports of having a lower QOL after disease onset [32]. We also found that a high CT score on admission was a predictor of a low RCS score at the 6-month follow-up, but no such association was found at the 3-month follow-up. This can be explained by the slower RCS score recovery in those with a higher CT score than in those with a lower CT score, which widened the score difference between the two groups at 6 months (Figure 3b). These results suggest that the extent of COVID-19 pneumonia could be associated with the process of recovering from PASC. Although the details of the immunobiology associated with PASC are currently under investigation, aberrant innate immune stimulation during acute COVID-19 might be associated with PASC [33]. In particular, respiratory sequelae are thought to occur because of impairments in the airways owing to endothelial damage and the inflammatory reaction that occurs in the acute phase [34]. Patients with severe COVID-19 pneumonia are prone to respiratory sequelae, and it has been shown that these patients experience a prolonged impairment in terms of their lungs’ capacity to diffuse carbon monoxide 3–12 months after disease onset [35,36]. Previous studies have shown that chest CT scans are an important tool for diagnosing COVID-19 [37] and that they can indicate disease severity and provide a short-term prognosis [38,39]. The results of the present study suggest that patients with extensive COVID-19 pneumonia in the acute phase experience a prolonged decline in HRQOL related to social activities. Thus, evaluating the extent of COVID-19 pneumonia by conducting a chest CT scan on admission could be helpful in considering the long-term medical and social support needs of COVID-19 survivors.

## 7. Limitations

This study had several limitations. First, a reduction in the number of participants available between the recruitment and analysis phases occurred as a trade-off for greater data integrity; thus, there may have been selection bias. Participants were excluded because they provided inadequate responses to the SF-36 questionnaire (or failed to complete it whatsoever) or because they missed either the 3-month or 6-month follow-up appointment. Second, there were no baseline SF-36 or pulmonary function data; therefore, comparisons with pre-COVID-19 onset were impossible. Third, almost all the patients in this study were diagnosed with COVID-19 before vaccination began in Japan (only one patient included in the analysis was infected with COVID-19 after being vaccinated), which complicates comparisons with the sequelae of patients who were infected with more recent strains. Fourth, this study was designed to include only patients who had been hospitalized; therefore, the findings cannot be generalized to those with mild illness. Fifth, as this study was conducted in outpatient clinics where discharged patients were visited voluntarily, patients whose health condition improved after discharge may not have been included. Sixth, this study was not designed to measure pulmonary function tests at 3 months after onset. This is because the study started at the beginning of the pandemic, when there was still a lack of information about the duration of infectivity, and therefore, there was greater concern about the risk of transmission. Despite these various limitations, our in-depth evaluation of the trajectory of PASC, HRQOL, and associated examinations enabled us to clarify the interrelationships among these factors. This led us to elucidate the potential involvement of COVID-19 pneumonia in the prolonged decline in certain HRQOL factors associated with PASC.

## 8. Conclusions

Our systematic assessment of HRQOL after COVID-19 infection revealed decreased SF-36 scores related to social activities in participants experiencing PASC, especially those with respiratory sequelae. A decrease in SF-36 scores related to social activities was also apparent, and this decrease was associated with chronic respiratory sequelae. Particular attention should be given to patients with extensive COVID-19 pneumonia observable on chest CT scans in the acute phase because this population may be more likely to experience a prolonged decline in HRQOL related to social activities.

## Figures and Tables

**Table 1:** Patient characteristics (n = 74).

Parameter	n = 74	Parameter	n = 74
Age group		Treatment received during hospital stay	65 (88%)
65 years	40 (54%)	Corticosteroids	48 (65%)
≥65 years	34 (46%)	Remdesivir	25 (34%)
Sex		Tocilizumab	5 (7%)
Male	54 (73%)	Baricitinib	2 (3%)
Female	20 (27%)	Medical equipment	
BMI		Supplemental Oxygen	59 (80%)
<25 (kg/m^2^)	40 (54%)	High-flow nasal cannula	2 (3%)
≥25 (kg/m^2^)	27 (36%)	Intubation	14 (19%)
Unknown	7 (10%)	ECMO	1 (1%)
Smoking history Yes	48 (65%)	Length of hospital stay, days	19.8 (13.8)
No	26 (35%)		
Comorbidity	54 (73%)		
Hypertension	35 (47%)		
Diabetes	18 (24%)		
Cardiovascular disease	11 (15%)		
Cerebrovascular disease	6 (8%)		
Malignant tumor	3 (4%)		
Autoimmune disease	3 (4%)		
Respiratory disease	16 (22%)		
Interstitial lung disease	3 (4%)		
COPD	9 (12%)		
Bronchial asthma	7 (9%)		

Note: Categorical variables are expressed as numbers (percentages), and continuous variables are expressed as mean (standard deviation). Respiratory disease includes interstitial lung disease, COPD, or bronchial asthma. Abbreviations: BMI, body mass index; COPD, chronic obstructive pulmonary disease; ECMO, Extracorporeal Membrane Oxygenation.

**Table 2:** SF-36 scores normalized by the Japanese national standards at each time point.

	3 Months	6 Months
SF-36 Scale	Mean score (±SD)	Mean score (±SD)
PF	46.6 ± 9.7	47.9 ± 8.1
RP	43.9 ± 13.1	46.7 ± 11.7
BP	49.8 ± 11.9	48.8 ± 11.1
GH	51.2 ± 7.6	51.9 ± 8.01
VT	54.3 ± 10.0	53.8 ± 10.1
SF	42.8 ± 14.3	47.6 ± 11.4
RE	45.0 ± 12.8	48.1 ± 11.3
MH	52.6 ± 9.1	53.9 ± 8.71
PCS	48.4 ± 11.0	47.5 ± 9.8
MCS	56.1 ± 8.5	55.5 ± 9.1
RCS	40.7 ± 13.9	45.7 ± 12.3

Note: Scores are expressed as mean ± standard deviations (SD). Abbreviations: PF: physical function, RP: role physical, BP: bodily pain, GH: general health, VT: vitality, SF: social functioning, RE: role emotional, MH: mental health, PCS: physical component summary, MCS: mental component summary, RCS: role/social component summary.

**Table 3:** Association between PASC, pulmonary function tests, and RCS score.

	Role/Social Component Summary Score							
	3 Months				6 Months			
	Coefficient	95% C.I.		*p*-Value	Coefficient	95% C.I.		*p*-Value
**PASC at each time points (yes/no)**								
any symptoms	−4.76	−11.3	1.78	0.151	−5.52	−11.91	0.87	0.090
respiratory symptoms	−6.99	−13.54	−0.44	**0.037**	−5.74	−12.35	0.88	0.088
dyspnea	1.20	−6.76	9.14	0.766	−7.57	−16.23	1.10	0.086
cough	−6.86	−16.3	2.58	0.152	−2.94	−15.71	9.82	0.647
fatigue	−8.85	−20.67	2.96	0.140	−4.42	−14.25	5.40	0.372
numbness	−1.70	−16.17	12.78	0.816	2.50	−10.27	15.28	0.697
joint pain	−13.47	−33.41	6.48	0.182	2.88	−22.14	27.90	0.819
olfactory disorder	13.82	−6.11	33.75	0.171	-	-	-	-
**Pulmonary Function Test**								
FVC (L)	-	-	-	-	3.59	0.40	6.78	**0.028**
%FVC (%)	-	-	-	-	0.19	0.01	0.38	**0.045**
FEV1 (L)	-	-	-	-	3.99	0.24	7.73	**0.037**
FEV1/FVC (%)	-	-	-	-	−0.07	−0.31	0.17	0.554

Note: Comparisons were determined by simple regression analysis. Dyspnea, cough, or fatigue were defined as respiratory symptoms. Abbreviations: PASC, post-acute sequelae of SARS-CoV-2 infection; FVC, forced vital capacity; FEV1, forced expiratory volume in 1 s. “-” indicates no comparative patients. Statistically significant differences (*p* < 0.05) are in bold.

**Table 4:** Association of patient backgrounds, status during hospitalization, and RCS score.

	Role/Social Component Summary Score							
	3 Months				6 Months			
	Coefficient	95% C.I.		*p*-Value	Coefficient	95% C.I.		*p*-Value
**Background (yes/no)**								
Age ≥ 65 (years old)	−7.74	−14.00	−1.45	**0.016**	−6.91	−12.50	−1.32	**0.016**
Sex, male	−4.61	−11.90	2.68	0.212	−0.32	−6.84	6.21	0.923
BMI ≥ 25 (kg/m^2^)	2.00	−5.31	9.31	0.586	−3.25	−9.58	3.07	0.308
Smoking history	0.85	−6.01	7.71	0.806	−3.69	−9.70	2.33	0.226
Any comorbidities	−3.60	−3.59	−10.90	0.332	−6.23	−12.58	0.13	0.055
Respiratory disease	−7.42	−15.18	0.34	0.061	−1.19	−8.22	5.84	0.736
**Status during hospitalization (yes/no)**								
Treatment								
Corticosteroids	0.73	−6.13	7.59	0.830	−1.39	−7.46	4.67	0.648
Remdesivir	2.20	−4.70	9.11	0.526	0.81	−5.32	6.94	0.793
Tocilizumab	−5.13	−18.12	7.86	0.430	−5.06	−16.52	6.39	0.381
Baricitinib	11.72	−8.55	31.99	0.250	1.32	−16.71	19.36	0.884
Medical equipment								
Supplemental Oxygen	−7.80	−15.73	0.14	0.054	−5.76	−12.83	1.31	0.109
High-flow nasal cannula	−26.57	−45.78	−7.37	**0.007**	−6.74	−24.50	11.01	0.452
Intubation	−6.88	−15.08	1.33	0.099	1.34	−6.04	8.73	0.718
ECMO	−2.03	−30.39	26.33	0.887	2.90	−22.14	27.91	0.819
CT score (≥10 points) on admission	−4.08	−10.87	2.70	0.233	−7.76	−13.88	−1.65	**0.014**

Note: Comparisons were determined by a simple regression analysis. Respiratory disease includes interstitial lung disease, COPD, or bronchial asthma. Abbreviations: BMI, body mass index; ECMO Extracorporeal Membrane Oxygenation. Statistically significant differences (*p* < 0.05) are in bold.

**Table 5:** Multivariate analysis results of factors predicting low RCS.

	3 Months				6 Months			
	Coefficient	95% C.I.		*p*-Value	Coefficient	95% C.I.		*p*-Value
Age ≥ 65 (years old)	−6.39	−13.69	0.91	0.085	−6.08	−12.89	0.72	0.078
Sex, male	−1.22	−9.26	6.81	0.762	1.47	−6.02	8.96	0.695
Smoking history	3.80	−3.52	11.12	0.303	−2.75	−9.57	4.08	0.424
Intubation	−10.00	−19.06	−0.95	**0.031**	2.84	−5.60	11.28	0.503
Respiratory disease	−5.91	−14.39	2.58	0.169	4.65	−3.26	12.55	0.244
CT score (≥10 points)	0.59	−6.74	7.93	0.872	−8.72	−15.55	−1.88	**0.013**

Note: Analyses were conducted using a multiple regression model. RCS score was an objective variable. The following dichotomized explanatory variables were entered into the model simultaneously: age (older than 65 years), sex (male), smoking history, intubation, respiratory disease, and CT score (cut-off by median value, ≥10 points). Abbreviation: RCS, role/social component summary. Statistically significant differences (*p* < 0.05) are in bold.

## Data Availability

The datasets used and analyzed in this study are available from the corresponding author upon reasonable request. The data are not publicly available due to privacy reasons.

## References

[1] COVID-19 Dashboard by the Center for Systems Science and Engineering (CSSE) at Johns Hopkins University (JHU). https://coronavirus.jhu.edu/map.html.

[2] Cao B., Wang Y., Wen D., Liu W., Wang J., Fan G., Ruan L., Song B., Cai Y., Wei M. (2020). A Trial of Lopinavir-Ritonavir in Adults Hospitalized with Severe COVID-19. N. Engl. J. Med..

[3] Wang C., Liu B., Zhang S., Huang N., Zhao T., Lu Q.B., Cui F. (2022). Differences in incidence and fatality of COVID-19 by SARS-CoV-2 Omicron variant versus Delta variant in relation to vaccine coverage: A world-wide review. J. Med. Virol..

[4] World Health Organization A Clinical Case Definition of Post COVID-19 Condition by a Delphi Consensus. https://www.who.int/publications/i/item/WHO-2019-nCoV-Post_COVID-19_condition-Clinical_case_definition-2021.1.

[5] Nalbandian A., Sehgal K., Gupta A., Madhavan M.V., McGroder C., Stevens J.S., Cook J.R., Nordvig A.S., Shalev D., Sehrawat T.S. (2021). Post-acute COVID-19 syndrome. Nat. Med..

[6] Huang C., Huang L., Wang Y., Li X., Ren L., Gu X., Kang L., Guo L., Liu M., Zhou X. (2021). 6-month consequences of COVID-19 in patients discharged from hospital: A cohort study. Lancet.

[7] Tsampasian V., Elghazaly H., Chattopadhyay R., Debski M., Naing T.K.P., Garg P., Clark A., Ntatsaki E., Vassiliou V.S. (2023). Risk Factors Associated with Post-COVID-19 Condition: A Systematic Review and Meta-analysis. JAMA Intern. Med..

[8] Tarraso J., Safont B., Carbonell-Asins J.A., Fernandez-Fabrellas E., Sancho-Chust J.N., Naval E., Amat B., Herrera S., Ros J.A., Soler-Cataluna J.J. (2022). Lung function and radiological findings 1 year after COVID-19: A prospective follow-up. Respir. Res..

[9] Centers for Disease Control and Prevention Health-Related Quality of Life (HRQOL) Concepts. https://www.cdc.gov/hrqol/concept.htm.

[10] Ware J.E., Sherbourne C.D. (1992). The MOS 36-item short-form health survey (SF-36). I. Conceptual framework and item selection. Med. Care.

[11] Poudel A.N., Zhu S., Cooper N., Roderick P., Alwan N., Terrant C., Ziauddeen N., Yao G.L. (2021). Impact of Covid-19 on health-related quality of life of patients: A structured review. PLoS ONE.

[12] O’Brien K., Townsend L., Dowds J., Bennan C., Nadarajan P., Kent B., Murphy N., Sheill G., Martin-Loeches I., Guinan E. (2022). 1-year quality of life and health-outcomes in patients hospitalised with COVID-19: A longitudinal cohort study. Respir. Res..

[13] Magdy D.M., Metwally A., Tawab D.A., Hassan S.A., Makboul M., Farghaly S. (2022). Long-term COVID-19 effects on pulmonary function, exercise capacity, and health status. Ann. Thorac. Med..

[14] Chen K.-Y., Li T., Gong F.-H., Zhang J.-S., Li X.-K. (2020). Predictors of Health-Related Quality of Life and Influencing Factors for COVID-19 Patients, a Follow-Up at One Month. Front. Psychiatry.

[15] Muñoz-Corona C., Gutierrez-Canales L.G., Ortiz-Ledesma C., Martinez-Navarro L.J., Marcias A.E., Scavo-Montes D.A., Guani-Guerra E. (2022). Quality of life and persistence of COVID-19 symptoms 90 days after hospital discharge. J. Int. Med. Res..

[16] Segura-Ortí E., Martinez-Olmos F.J., Rodenas-Pascual A., Guillem-Gimenez E., Vercher-Narbona V., Pinon-Ruiz M.J., Garcia-Testal A. (2022). Impact of COVID-19 Pandemic on Health-Related Quality of Life and Physical Activity of Patients in Hemodialysis. Healthcare.

[17] van den Borst B., Peter J.B., Brink M., Schoon Y., Bleeker-Rovers C.P., Schers H., van Hees H.W.H., van Helvoort H., van den Boogaard M., van der Hoeven H. (2021). Comprehensive Health Assessment 3 Months After Recovery from Acute Coronavirus Disease 2019 (COVID-19). Clin. Infect. Dis..

[18] Kersten J., Wolf A., Hoyo L., Hull E., Tadic M., Andreß S., d’Almeida S., Scharnbeck D., Roder E., Beschoner P. (2022). Symptom burden correlates to impairment of diffusion capacity and exercise intolerance in long COVID patients. Sci. Rep..

[19] Suzukamo Y., Fukuhara S., Green J., Kosinski M., Gandek B., Ware J.E. (2011). Validation testing of a three-component model of Short Form-36 scores. J. Clin. Epidemiol..

[20] Fukuhara S., Bito S., Green J., Hsiao A., Kurokawa K. (1998). Translation, adaptation, and validation of the SF-36 Health Survey for use in Japan. J. Clin. Epidemiol..

[21] Fukuhara S., Ware J.E., Kosinski M., Wada S., Gandek B. (1998). Psychometric and clinical tests of validity of the Japanese SF-36 Health Survey. J. Clin. Epidemiol..

[22] Chang Y.-C., Yu C.-J., Chang S.-C., Galvin J.-R., Liu H.-M., Hsiao C.-H., Kuo P.-H., Chen K.-Y., Franks T.-J., Huang K.-M. (2005). Pulmonary sequelae in convalescent patients after severe acute respiratory syndrome: Evaluation with thin-section CT. Radiology.

[23] Graham B.L., Steenbruggen I., Miller M.R., Barjaktarevic I.Z., Cooper B.G., Hall G.L., Hallstrand T.S., Kaminsky D.A., McCarthy K., McCormack M.C. (2019). Standardization of Spirometry 2019 Update. An Official American Thoracic Society and European Respiratory Society Technical Statement. Am. J. Respir. Crit. Care Med..

[24] Graham B.L., Brusasco V., Burgos F., Cooper B.G., Jensen R., Kendrick A., Maclntyre N.R., Thompson B.R., Wanger J. (2017). 2017 ERS/ATS standards for single-breath carbon monoxide uptake in the lung. Eur. Respir. J..

[25] Kanda Y. (2013). Investigation of the freely available easy-to-use software “EZR” for medical statistics. Bone Marrow Transpl..

[26] Sugiyama A., Miwata K., Kitahara Y., Okimoto M., Abe K., Bunthen E., Ouoba S., Akita T., Tanimine N., Ohdan H. (2022). Long COVID occurrence in COVID-19 survivors. Sci. Rep..

[27] Terai H., Ishii M., Takemura R., Namkoong H., Shimamoto K., Masaki K., Tanosaki T., Chubachi S., Matsuyama E., Hayashi R. (2023). Comprehensive analysis of long COVID in a Japanese nationwide prospective cohort study. Respir. Investig..

[28] Rodríguez-Galán I., Albaladejo-Blazquez N., Ruiz-Robledillo N., Pascual-Liedo J.F., Ferrer-Cascales R., Gil-Carbonell J. (2022). Impact of COVID-19 on Health-Related Quality of Life: A Longitudinal Study in a Spanish Clinical Sample. Int. J. Environ. Res. Public Health.

[29] Rass V., Ianosi B.A., Zamarian L., Beer R., Sahanic S., Lindner A., Kofler M., Schiefecker A.J., Mahlknecht P., Heim B. (2022). Factors associated with impaired quality of life three months after being diagnosed with COVID-19. Qual. Life Res..

[30] Mahler D.A., Mackowiak J.I. (1995). Evaluation of the short-form 36-item questionnaire to measure health-related quality of life in patients with COPD. Chest.

[31] Martinez T.Y., Pereira C.A., dos Santos M.L., Ciconelli R.M., Guimaraes S.M., Martinez J.A. (2000). Evaluation of the short-form 36-item questionnaire to measure health-related quality of life in patients with idiopathic pulmonary fibrosis. Chest.

[32] Malik P., Patel K., Pinto C., Jaiswal R., Tirupathi R., Pillai S., Patel U. (2022). Post-acute COVID-19 syndrome (PCS) and health-related quality of life (HRQoL)-A systematic review and meta-analysis. J. Med. Virol..

[33] Merad M., Blish C.A., Sallusto F., Iwasaki A. (2022). The immunology and immunopathology of COVID-19. Science.

[34] Wang F., Kream R.M., Stefano G.B. (2020). Long-Term Respiratory and Neurological Sequelae of COVID-19. Med. Sci. Monit..

[35] Bellan M., Baricich A., Patrucco F., Zeppegno P., Gramaglia C., Balbo P.E., Carriero A., Amico C.S., Avanzi G.C., Barini M. (2021). Long-term sequelae are highly prevalent one year after hospitalization for severe COVID-19. Sci. Rep..

[36] Froidure A., Mahsouli A., Liistro G., De Greef J., Belkhir L., Gerad L., Bertrand A., Koenig S., Pothen L., Yildiz H. (2021). Integrative respiratory follow-up of severe COVID-19 reveals common functional and lung imaging sequelae. Respir. Med..

[37] Alsharif W., Qurashi A. (2021). Effectiveness of COVID-19 diagnosis and management tools: A review. Radiography.

[38] Francone M., Iafrate F., Masci G.M., Coco S., Cilia F., Manganaro L., Panebianco V., Andreoli C., Colaiacomo M.C., Zingaropoli M.A. (2020). Chest CT score in COVID-19 patients: Correlation with disease severity and short-term prognosis. Eur. Radiol..

[39] González J., Benitez I.D., Carcoma P., Santisteve S., Monge A., Moncusi-Moix A., Gort-Paniello C., Pinilla L., Carratala A., Zuil M. (2021). Pulmonary Function and Radiologic Features in Survivors of Critical COVID-19: A 3-Month Prospective Cohort. Chest.

